# Supplementary material for: Identification of photoperiod-induced specific miRNAs in the adrenal glands of Sunite sheep (Ovis aries)
Source: Front Vet Sci. 2022 Jul 22;9:888207. doi: 10.3389/fvets.2022.888207 (PMC9354845; doi:10.3389/fvets.2022.888207)
Supplement: Supplementary file 1 [file Data_Sheet_1.ZIP › Supplementary file/miRDeep2 file.zip!/miRDeep2 file/Know_Structure/SPLP42A2/oar-mir-409.pdf]

miRBase precursor : oar-mir-409
Total read count : 12981
oar-miR-409-5p read count : 2905
oar-miR-409-3p read count : 10066
remaining reads : 10

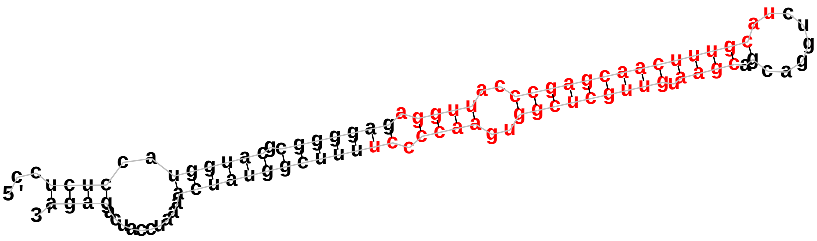

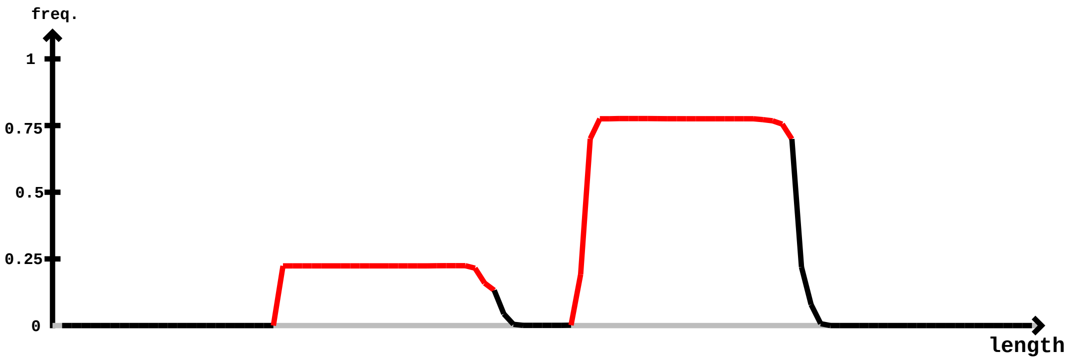


```
                                oar-miR-409-5p
                                                         oar-miR-409-3p
5'- ccucuccaugguacgcggggagagguuacccgagcaacuuugcaucuggacgacgaauguugcucggugaaccccuuuucgguaucaaaauccaucagaga   -3'    exp
    .((((...((((((.(((((((.((((..((((((((((((((........).)))).)))))))))..)))).))))))))))))).........)))).   reads     mm        sample
    ......................agguuacccgagcaacuuu............................................................   1         0         seq
    ......................agguuacccgagcaacuuug...........................................................   120       0         seq
    ......................agguuacccgagcaacuuugc..........................................................   727       0         seq
    ......................agguuacccgagcaacuuugca.........................................................   348       0         seq
    ......................agguuacccgagcaacuuugcau........................................................   1148      0         seq
    ......................agguuacccgagcaacuuugcauc.......................................................   509       0         seq
    ......................agguuacccgagcaacuuugcaucu......................................................   51        0         seq
    .......................gguuacccgagcaacuuugca.........................................................   1         0         seq
    ......................................uuugcaucuggacgacgaauguu........................................   4         0         seq
    ......................................uuugcaucuggacgacgaauguug.......................................   3         0         seq
    .......................................uugcaucuggacgacgaauguug.......................................   1         0         seq
    .......................................uugcaucuggacgacgaauguugcu.....................................   1         0         seq
    ..............................................uggacgacgaauguugcucggugaaccccu.........................   1         0         seq
    ....................................................acgaauguugcucggugaac.............................   5         0         seq
    ....................................................acgaauguugcucggugaacc............................   2         0         seq
    ....................................................acgaauguugcucggugaaccccu.........................   1         0         seq
    .....................................................cgaauguugcucggugaa..............................   2         0         seq
    .....................................................cgaauguugcucggugaac.............................   27        0         seq
    .....................................................cgaauguugcucggugaacc............................   33        0         seq
    .....................................................cgaauguugcucggugaaccc...........................   66        0         seq
    .....................................................cgaauguugcucggugaacccc..........................   268       0         seq
    .....................................................cgaauguugcucggugaaccccu.........................   1555      0         seq
    .....................................................cgaauguugcucggugaaccccuu........................   428       0         seq
    .....................................................cgaauguugcucggugaaccccuuu.......................   85        0         seq
    .....................................................cgaauguugcucggugaaccccuuuu......................   11        0         seq
    ......................................................gaauguugcucggugaac.............................   10        0         seq
    ......................................................gaauguugcucggugaacc............................   21        0         seq
    ......................................................gaauguugcucggugaaccc...........................   86        0         seq
    ......................................................gaauguugUucggugaaccc...........................   2         1         seq
    ......................................................gaauguugcucggugaacccc..........................   414       0         seq
    ......................................................gaauguugcucggugaaccccu.........................   4017      0         seq
    ......................................................gaauguugcucggugaaccccuu........................   1253      0         seq
    ......................................................gaauguugcucggugaaccccuuu.......................   739       0         seq
    ......................................................gaauguugcucggugaaccccuuuu......................   60        0         seq
```

```
                      oar-miR-409-5p
                                                     oar-miR-409-3p
ccucuccaugguacgcggggagagguuacccgagcaacuuugcaucuggacgacgaauguugcucggugaaccccuuuucgguaucaaaauccaucagaga

.......................................................aauguugcucggugaac.............................    1    0    seq
.......................................................aauguugcucggugaaccc...........................    8    0    seq
.......................................................aauguugcucggugaacccc..........................    43   0    seq
.......................................................aauguugcucggugaaccccu.........................    658  0    seq
.......................................................aauguugcucggugaaccccuu........................    143  0    seq
.......................................................aauguugcucggugaaccccuuu.......................    101  0    seq
.......................................................aauguugcucggugaaccccuuuu......................    16   0    seq
........................................................auguugcucggugaaccccuuuu......................    1    0    seq
.........................................................uguugcucggugaaccccuuu.......................    6    0    seq
.........................................................uguugcucggugaaccccuuuu......................    3    0    seq
.........................................................uguugcucggugaaccccuuuuc.....................    1    0    seq
```
